# Evaluation of a nationwide whole-school approach to mental health and well-being in 40 149 Australian secondary school students: cluster quasi-experimental study

**DOI:** 10.1192/bjo.2024.843

**Published:** 2025-03-11

**Authors:** Roshini Balasooriya Lekamge, Md Nazmul Karim, Leo Chen, Dragan Ilic

**Affiliations:** School of Public Health and Preventative Medicine, Monash University, Melbourne, Australia; Department of Psychiatry, School of Translational Medicine, Monash University, Melbourne, Australia; Alfred Mental and Addiction Health, Alfred Health, Melbourne, Australia

**Keywords:** Mental health and mental disorders, adolescence, secondary schools, whole-school approach, Health-Promoting Schools Framework

## Abstract

**Background:**

Adolescence is the peak life stage for the development of mental illness. Whole-school approaches to mental health and well-being, modelled on the World Health Organization’s Health-Promoting Schools Framework, hold vast potential in this developmentally sensitive period. However, the evidence base for these interventions is inconclusive.

**Aims:**

Our study examines the effectiveness of The Resilience Project School Partnership Program, a whole-school intervention involving students, teachers and parents, centred around concepts of gratitude, empathy, emotional literacy and mindfulness.

**Methods:**

A quasi-experimental study with an intervention and a control arm was used to evaluate the programme in 40 149 students across 102 schools in 2023. Data collected included sociodemographic information and outcomes derived from validated scales, comprising life satisfaction, hope, coping skills, anxiety and depression. Intervention schools were stratified by the number of years they had implemented the programme, and mixed-effects regression models were used to evaluate the programme.

**Results:**

After adjusting for confounders, participants at schools who had been implementing the programme for 6 years or longer demonstrated significantly better outcomes across all five domains (life satisfaction: *B* = 0.627, 95% CI 0.465–0.789; hope: *B* = 2.135, 95% CI 0.895–3.347; coping skills: *B* = 0.438, 95% CI 0.250–0.625; anxiety: odds ratio = 0.658, 95% CI 0.559–0.774; depression: odds ratio = 0.534, 95% CI 0.459–0.620). Only depression was significantly lower among participants at schools in their fourth or fifth year of implementing the programme (odds ratio = 0.941, 95% CI 0.935–0.948).

**Conclusions:**

Our findings indicate that whole-school interventions may require long-term investment to realise their potential and highlight implementation duration as an important consideration for future evaluations of whole-school interventions.

A mental health crisis is emerging among Australian youth and is reflective of a global trend.^1^ Adolescence is a time of particular vulnerability to poor mental health, with a recent meta-analysis identifying the peak age at onset for mental illness to be 14.5 years.^2^ An Australian-based national study found that 32.4% of Australian males and 45.5% of Australian females aged 16–24 years had experienced a mental illness in the preceding 12 months, the highest prevalence in any age category.^3^

## Whole-school approaches to mental health and well-being

**Fig. 1:**
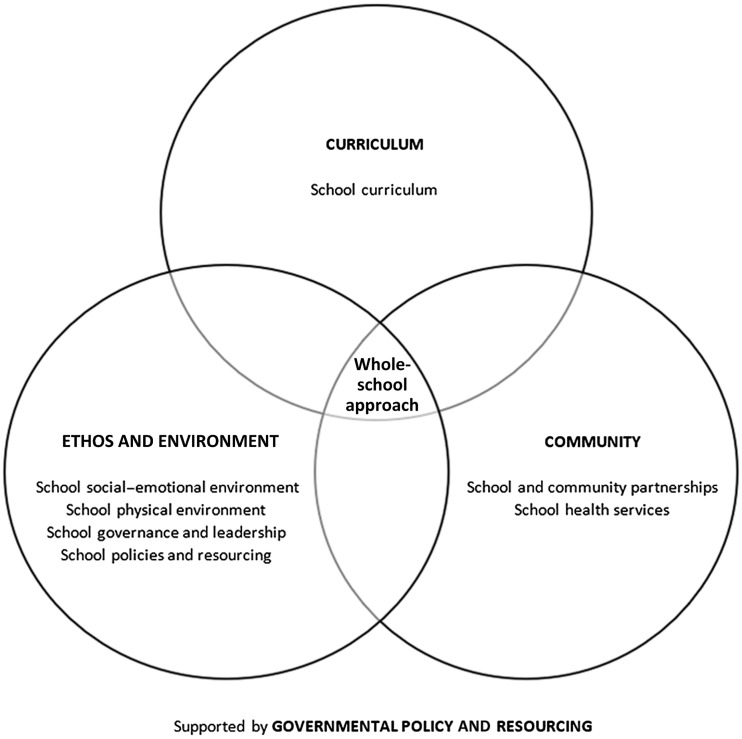
Global standards of the Health-Promoting Schools Framework (reproduced from Lekamge et al.^11^ under the Creative Commons CC BY license).

Schools have long been considered to be influential settings for promotion of mental health and well-being in adolescents, as most adolescents spend a substantial proportion of their time in school.^4^ Whole-school interventions are particularly attractive by virtue of their holistic focus, which shifts attention away from individual behaviour change and towards a socio-ecological approach to health promotion.^5,6^ Whole-school approaches are multicomponent interventions modelled on the World Health Organization’s Health-Promoting Schools Framework. This framework was updated in 2021 to comprise eight global standards,^7^ which can be categorised into four different levels (Fig. 1). Interventions are generally classified as whole-school if they incorporate a component addressing each of the following levels: curriculum, ethos and environment and community.^8,9^ Whole-school interventions are theoretically more likely to improve adolescent mental health and well-being than other forms of school-based approaches.^6,9,10^ This has been attributed to their use of a holistic, systems-based approach and involvement of the key stakeholders in an adolescent’s life, including their peers, parents and teachers.

Despite a strong theoretical basis for whole-school approaches to mental health and well-being, meta-analyses exploring their effectiveness have provided inconclusive evidence.^8,12,13^ A 2015 meta-analysis concluded that although whole-school approaches significantly reduced the odds of tobacco use or being bullied among school-aged populations, there was no evidence of effectiveness in other domains, including alcohol or drug use, aggression and bullying others and mental health outcomes. This inconclusive evidence base is commonly attributed to the complexity of evaluating whole-school interventions^14^ and the challenges encountered in their implementation.^13^

A domain that has received comparatively less attention is implementation duration. Public health initiatives adopting a socio-ecological approach address more than simply individual-level factors affecting behavioural and health outcomes. Realisation of such systems-level change is likely to require time. Despite this, a systematic review of community-based health promotion programmes found that these typically lasted for only 2 to 3 years, with review authors calling into question whether such short durations were sufficient to demonstrate impact on behavioural and health outcomes.^15^ A 2015 meta-analysis similarly found that whole-school approaches to mental health and well-being among secondary school-aged populations could involve interventions as short as 15 weeks, with the vast majority of interventions implemented for 2 to 3 years.^8^ Given the complex changes that whole-school approaches aspire to achieve, including changes to the school ethos and environment, as well as school relationships with parents and the local community, it is plausible that they may require long-term investment to realise their potential. It is thus important to investigate whether the evidence base surrounding whole-school approaches to mental health and well-being is inconclusive because of programme ineffectiveness, or whether temporal investment represents a factor key to programme success.

## Current study

While whole-school approaches to mental health and well-being offer a promising strategy in improving adolescent mental health, the evidence base for these interventions is inconclusive.^8^ There is a particular need to investigate the impact of implementation duration on the effectiveness of whole-school approaches, as this may reveal valuable insights for key stakeholders, including schools and public health and policy makers. Here, we evaluate the Resilience Project School Partnership Program, a whole-school intervention promoting mental health and well-being in Australia.^16^ The programme involves students, teachers and parents and is centred around the teaching and practice of four core elements: gratitude, empathy, emotional literacy and mindfulness (GEEM). In its 10 years of implementation, the intervention has reached more than 1500 schools and early learning centres, and in 2023, the programme was implemented in more than 800 primary and secondary schools across Australia. The objective of this study was to assess whether the intervention group demonstrated better mental health outcomes across life satisfaction, hope, coping skills, anxiety and depression than the control group. A unique feature of this study was that the intervention schools included had implemented the programme for 2–8 years, enabling us to explore whether schools with longer implementation duration exhibited better mental health outcomes.

## Methods

We conducted a quasi-experimental study that consisted of an experimental and control arm, with post-test data but no pre-test data,^17^ consistent with study design B1 as described by Harris et al.^17^ The experimental arm consisted of a whole-school intervention delivered by the Resilience Project.^18^ Outcomes were measured independently by Resilient Youth Australia,^19^ annually, via an online, user-report system. To partake in the intervention or survey, schools had to enter into a service agreement with the Resilience Project and/or Resilient Youth Australia. Assignment of schools to conditions was by administrator selection, whereby a school’s executive team self-selected their schooling cluster into the experimental or control condition at the commencement of the study.^20^ Any school that was in at least its second year of implementing the programme, that was implementing the programme for all grade levels, and that agreed at the outset to having students participate in data collection was included in the intervention group. The control group consisted of schools that agreed to having their students undertake data collection but were not actively implementing the programme and had not previously implemented the programme.

The authors assert that all procedures contributing to this work comply with the ethical standards of the relevant national and institutional committees on human experimentation and with the Helsinki Declaration of 1975, as revised in 2008. All procedures involving human participants were approved by the Monash University Human Research Ethics Committee (project ID: 37824). Informed consent by participants was deemed not necessary by the Research Ethics Committee owing to the anonymous nature of the data collected.

### Participants

Participants were required to meet all aspects of the eligibility criteria, including: (a) enrolment in grades 7 to 12 in an Australian secondary school in 2023; (b) age 11 to 19 years in 2023; and (c) attending a school that was in at least its second year of implementing the programme and that offered the programme to all grade levels, or attending a school that was assigned as part of the control group. The sociodemographic characteristics of the sample are detailed in Table 2.

### Intervention

The programme consisted of a whole-school approach to cultivating happiness and resilience in young people. At the curriculum level, the programme comprised 50 year-level-specific lessons to facilitate students’ understanding and practice of GEEM. At the ethos and environment level, schools were provided with activities to foster GEEM outside the formal curriculum; resources to embed GEEM chat boards and gratitude boards into the physical environment; professional development opportunities to build staff capacity for implementing the programme; and resources to facilitate the practice of GEEM as a staff cohort. At the community level, parents and carers were provided with access to an online hub comprising informational videos, family well-being activities and links to additional resources. The programme is embedded among governmental initiatives to promote mental health and well-being in schools, with Be You and the Schools Mental Health Menu serving as examples of national and subnational initiatives, respectively.^16,21^ Each intervention school was allocated a Schools Partnership Manager to assist with programme implementation. A detailed description of the programme in accordance with the TIDieR checklist^22^ and sample lesson plans are provided in Supplementary Material 1 and 2, available at https://doi.org/10.1192/bjo.2024.843.

### Data sources

Data were collected from January to December in 2023 through an online survey administered in schools. The Resilience Survey was co-developed with the School of Psychology, Social Work and Social Policy at the University of South Australia. The 60-item survey incorporates five scientifically validated measures: the Cantril Self-Anchoring Scale (CSAS), Children’s Hope Scale (CHS), Coping Strategies Inventory (CSI) Disengagement Subscale, Generalised Anxiety Disorder (GAD-2) and Patient Health Questionnaire (PHQ-2). We evaluated the programme’s effect based on these outcome measures. Survey questions from these measures are included in Supplementary Material 3.

#### Cantril Self-Anchoring Scale (adapted)

The adapted CSAS is a single-item rating scale in which students rank their life satisfaction from 1 to 8, with a higher score indicating greater life satisfaction. An 11-point version of the CSAS has demonstrated reliability and convergent validity with six previously validated measures of subjective well-being in a sample of 7670 adolescents in Scotland.^23^ The scale used here was adapted in that the wording was simplified and an eight-point version was used (Supplementary Material 3).

#### Children’s Hope Scale

The CHS is a six-item scale, with each item recorded on a six-point Likert scale. Scores are summed from the six items, producing a total score out of 36, with a higher aggregate score indicating greater hope. The CHS has demonstrated reliability, convergent and discriminant validity in studies comprising children and adolescents.^24,25^ Cronbach’s alpha in our sample was 0.897 (95% CI 0.895–0.899).

#### Coping Strategies Inventory Disengagement Subscale (adapted)

The CSI disengagement subscale encompasses four items, with each item recorded on a four-point Likert scale. Items are summed to produce an overall score out of 16, with a higher aggregate score indicating greater coping skills. Studies have demonstrated the reliability^26^ and criterion and construct validity^27^ of the CSI. Hierarchical factor analysis has demonstrated two tertiary scales, one of which is the disengagement scale used here.^26^ The adapted version includes minor simplifications to the wording of the original disengagement subscale (Supplementary Material 3). Cronbach’s alpha in our sample was 0.523 (95% CI 0.515–0.530).

#### Generalised Anxiety Disorder

The GAD-2 is a two-item screening tool for generalised anxiety disorder, with each item recorded on a four-point Likert scale. The scores are first summed and then dichotomised, with an aggregate score of 3 or more demonstrating acceptable sensitivity, specificity and discriminant validity as a screening tool for generalised anxiety disorder.^28,29^ Here, a score of <3 was coded as 0 (indicating the absence of anxiety), and a score of ≥3 was coded as 1 (indicating the presence of anxiety). Cronbach’s alpha in our sample was 0.861 (95% CI 0.858–0.863).

#### Patient Health Questionnaire

The PHQ-2 is a two-item screening tool for major depressive disorder, with each item recorded on a four-point Likert scale. The scores are then summed, with a total of 3 or more demonstrating acceptable sensitivity, specificity and discriminant validity as a screening tool for major depressive disorder.^28,30^ A score of <3 was coded as 0 (indicating the absence of depression), and a score of ≥3 was coded as 1 (indicating the presence of depression). Cronbach’s alpha in our sample was 0.729 (95% CI 0.724–0.735).

### Statistical methods

Baseline sociodemographic characteristics of the intervention and control groups were compared using chi-squared tests for categorical variables and independent *t*-tests for numerical variables. For comparison of outcomes between intervention and control schools, intervention schools were first stratified into categories according to their duration of implementing the programme: (a) second or third year, (b) fourth or fifth year, (c) sixth, seventh or eighth year. Each stratum was then compared with the control group to assess whether differential outcomes were observed across the three strata. These strata were selected as a recent meta-analysis found that the majority of whole-school interventions promoting mental health and well-being were implemented by schools for 2–3 years,^8^ whereas 6+ years of implementation would result in a subset of students who had received the programme for their entire secondary school experience. Given that individual data were nested within schools, which themselves were nested within states, mixed-effects models were used to compare group differences in outcomes. The mixed-effects models used a fixed effect for group (intervention versus control), a random effect for school to account for clustering of responses within schools (level 2), and a random effect for state to account for clustering of responses within states (level 3). The effects of clustering were negligible at the state level, but apparent at the school level (Table 1). This indicates that the majority of variation occurred at the individual level.

**Table 1:** Intraclass coefficients at the school-level for outcome measures

Outcome	Intraclass coefficient
Life satisfaction	0.02
Hope	0.04
Coping skills	0.01
Anxiety	0.02
Depression	0.02

Mixed-effects linear regression was used for continuous outcomes (all of which were approximately normally distributed), and mixed-effects logistic regression was used for binary outcomes. The models used maximum likelihood estimation and robust standard errors. Confounders that were adjusted for as fixed effects in all mixed-effects models were grade, gender, socioeconomic and rurality status, with each treated as a categorical covariate. Socioeconomic and rurality status were categorised according to the Index of Relative Socio-economic Advantage and Disadvantage^31^ and Remoteness Area,^32^ respectively, based on the location of a participant’s school. All analyses were conducted using Stata Basic Edition Version 18 for Windows (StataCorp LLC, College Station, Texas, USA).

## Results

### Participant characteristics

A total of 40 149 students participated in the study, consisting of 18 875 participants from 55 schools assigned to the intervention group and 21 274 participants from 47 schools assigned to the control group (Table 2). The mean age was slightly lower in the intervention group than in the control group (14.11 *v*. 14.47 years, range 11–19). There was a higher proportion of male participants in the intervention compared with the control group (48.33% *v*. 37.14%), with roughly equivalent proportions of non-binary participants (3.86% *v*. 3.33%). The control group had a higher proportion of participants from schools in metropolitan areas (89.98% *v*. 42.17%) and the highest socioeconomic quintile (54.57% *v*. 16.31%).

**Table 2:** Sociodemographic variables of the intervention and control groups

Variable	Intervention	Control
Participants	18 875	21 274
Number of schools	55	47
Age in years, mean (s.d.)	14.11 (1.63)	14.47 (1.72)
Grade, *n* (%)		
7	3972 (21.04)	4348 (20.44)
8	3870 (20.50)	3730 (17.53)
9	3822 (20.25)	3486 (16.39)
10	3057 (16.20)	3752 (17.64)
11	2449 (12.97)	3580 (16.83)
12	1705 (9.03)	2378 (11.18)
Gender, *n* (%)		
Female	9023 (47.80)	12 664 (59.53)
Male	9123 (48.33)	7901 (37.14)
Non-binary	729 (3.86)	709 (3.33)
Rurality status, *n* (%)		
Metropolitan	7960 (42.17)	19 143 (89.98)
Inner regional	6209 (32.90)	1921 (9.03)
Outer regional	4311 (22.84)	198 (0.93)
Remote or very remote	395 (2.09)	12 (0.06)
Socioeconomic status, *n* (%)		
1 (least advantaged)	162 (0.86)	430 (2.02)
2	4100 (21.72)	6175 (29.03)
3	4322 (22.90)	2676 (12.58)
4	7213 (38.21)	384 (1.81)
5 (most advantaged)	3078 (16.31)	11 609 (54.57)

*P* < 0.001 for all *t*-tests and chi-squared analyses between intervention and control groups.

**Fig. 2:**
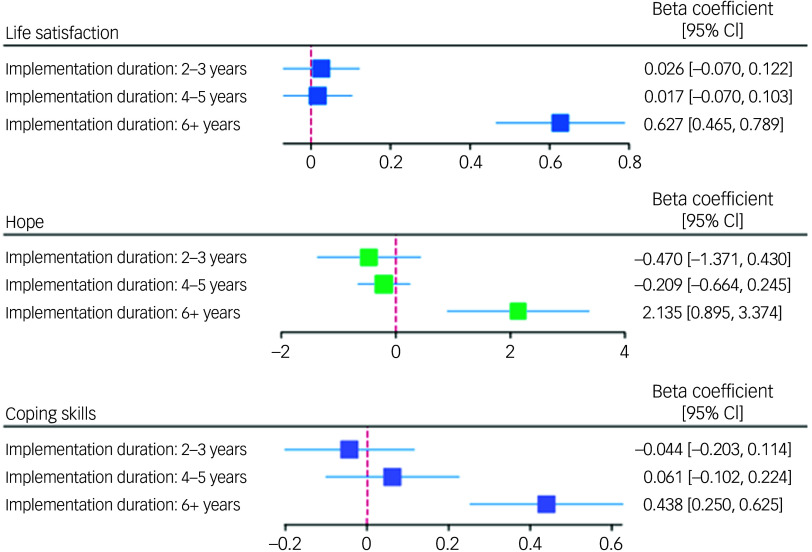
Graphical representation of linear mixed-effects model results. The reference for the null effect is represented by a maroon dashed line.

**Fig. 3:**
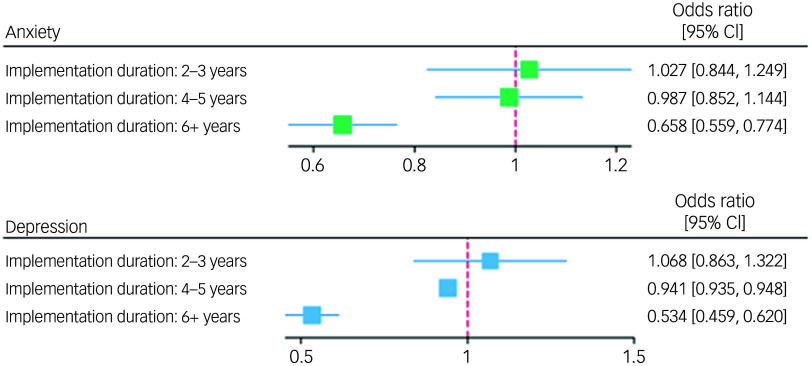
Graphical representation of logistic mixed-effects model results. The reference for the null effect is represented by a maroon dashed line.

Participants at schools in their second or third year of implementing the intervention did not show significant differences with respect to any of the five outcomes compared with the control group (Table 3). Participants at schools in their fourth or fifth year of implementing the intervention demonstrated significantly lower odds of depression than the control group (odds ratio = 0.941, 95% CI 0.935–0.948), but there were no significant differences in relation to other outcome domains. Participants at schools in at least their sixth year of implementing the intervention demonstrated significantly better scores across all outcome domains. This included higher scores across positive mental health outcomes (life satisfaction: *B* = 0.627, 95% CI 0.465–0.789; hope: *B* = 2.135, 95% CI 0.895–3.374; coping skills: *B* = 0.438, 95% CI 0.250–0.625) and significantly lower odds of mental illness than the control group (anxiety: odds ratio = 0.658, 95% CI 0.559–0.774; depression: odds ratio = 0.534, 95% CI 0.459–0.620). A graphical representation of these results is provided in Figs 2 and 3. The full mixed-effects models are included in Supplementary Material 4.

**Table 3:** Results from mixed-effects linear and logistic regression models, with intervention schools stratified by number of years implementing the programme (reference: control group); all results are adjusted for grade, gender, socioeconomic and rurality status

Outcome	2–3	4–5	6+
Life satisfaction, *B* (95% CI)	0.026 (–0.070, 0.122)	0.017 (–0.070, 0.103)	0.627 (0.465, 0.789)***
Hope, *B* (95% CI)	–0.470 (–1.371, 0.430)	–0.209 (–0.664, 0.245)	2.135 (0.895, 3.374)**
Coping skills, *B* (95% CI)	–0.044 (–0.203, 0.114)	0.061 (–0.102, 0.224)	0.438 (0.250, 0.625)***
Anxiety, OR (95% CI)	1.027 (0.844, 1.249)	0.987 (0.852, 1.144)	0.658 (0.559, 0.774)***
Depression, OR (95% CI)	1.068 (0.863, 1.322)	0.941 (0.935, 0.948)***	0.534 (0.459, 0.620)***

OR, odds ratio.

**P < 0.01, ***P < 0.001.

## Discussion

The findings of our nationwide evaluation indicate that participants receiving The Resilience Project School Partnership Program demonstrated consistently better mental health outcomes than the control group, provided that schools had invested in long-term implementation of the programme. Students at schools in at least their sixth year of implementing the programme demonstrated significantly better mental health outcomes than the control group, with this pattern consistent across all five outcome domains. A significant difference in outcomes emerged for participants at schools implementing the programme for 4 to 5 years, who demonstrated significantly lower odds for depression than the control group. By contrast, participants at schools implementing the programme for 2 to 3 years demonstrated no significant difference in outcomes compared with the control group.

A 2015 meta-analysis concluded that whole-school interventions in school-aged populations were ineffective with respect to the majority of mental health and well-being outcomes^8^, a surprising finding given the robust theoretical basis underlying whole-school interventions.^6,10^ One possible explanation for this is that the majority of whole-school interventions examined were implemented by schools for only 2 to 3 years,^8^ whereas our findings suggest that this could be too short a duration to achieve the complex systems-level changes that these interventions aspire to achieve. Evaluation of a whole-school intervention as ineffective after 2 to 3 years of implementation may not necessarily indicate programme ineffectiveness; rather, the programme may not have been implemented for long enough to achieve measurable change. Our findings indicate that long-term investment in whole-school interventions could lead to substantial benefit; for example, participants at schools implementing the programme for 6 years or longer had 34% lower odds for anxiety and 47% lower odds for depression than the control group. Given the manifold consequences of positive mental health and mental illness, long-term investment in these programmes holds vast potential to reduce the psychosocial and economic burden attributable to mental health.^21^

**Fig. 4:**
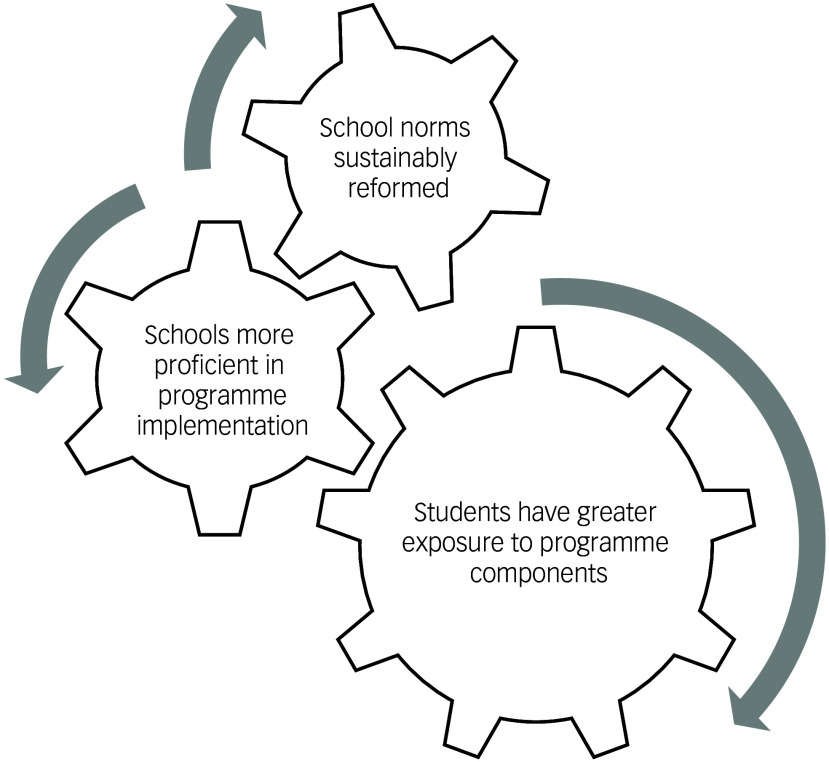
Conceptual model of why longer programme implementation may result in better effects.

Our study offers a conceptual model of why schools with longer implementation of the programme demonstrated better mental health outcomes, whereas those with shorter periods of implementation did not (Fig. 4). First, students at schools in at least their sixth year of implementing the programme are more likely to have had multi-year exposure to programme components. For example, a grade 12 student at a school in its sixth year of implementing the programme will have received the programme for their entire secondary school experience, provided they had remained at the same school. In 2023, the grade 7–12 apparent retention rate in Australia was 79.1%.^33^ Given that changes to one’s emotions, thoughts and behaviours are known to require time and iterative effort,^34^ longer exposure to the programme is more likely to be effective in modifying these faculties central to mental health and well-being.

Second, owing to their implementation in complex, multilevel systems and the involvement of multiple stakeholders, whole-school approaches commonly encounter challenges with implementation.^35^ It is likely that schools with longer implementation of whole-school interventions will become more proficient in their implementation and in troubleshooting the challenges that arise. Stronger implementation has subsequently been linked to better mental health outcomes, with a meta-analysis of school-based social and emotional learning programmes demonstrating that effect sizes were twice as high for interventions with high-quality implementation when compared to those with low-quality implementation.^13^

Finally, longer implementation of the programme is likely to translate into sustainable changes in school norms, for example, norms pertaining to the school ethos and environment. A 2019 umbrella review found that a highly demanding academic environment is a risk factor for poor adolescent mental health and well-being, whereas positive relations with teachers and peers, as well as perceptions of safety, belonging and connectedness, serve as protective factors.^10^ The influence of technology is also vital to consider, with a recent meta-analysis linking problematic smartphone use in youth to increased odds of stress, anxiety, depression and poor sleep and advocating that teachers help to limit exposure in school environments.^36^ Sustainable improvements to domains such as these can thus offer benefit to the mental health and well-being of old and new students alike. Nevertheless, although long-term investment in whole-school approaches can offer various advantages, a fundamental challenge lies in how to sustain resources. To overcome this, it is critical that stakeholders such as the government support schools in their ability to invest in whole-school initiatives in the long term, for example, through funding schemes and ensuring that schools are sufficiently staffed.

In placing the findings of our study in perspective, there are several limitations to consider. The first is the lack of randomisation inherent in the study design, which introduces the possibility of selection bias. However, the appropriateness of using randomised controlled trials (RCTs) to evaluate whole-school interventions is contentious. Some suggest that RCTs are incapable of capturing the complexity of systems-based approaches to public health issues,^10^ whereas others offer the counter-perspective that RCTs are an appropriate trial design and can accommodate the effects of local programme adaptation.^8^ A second limitation is the lack of pre-test results from the intervention and control group. The groups may differ from each other in systematic ways other than the intervention, and these differences may provide alternative explanations for the observed effect. For example, the control group was more heavily skewed towards female participants than the intervention group (59.53% *v*. 47.80%) and more heavily skewed towards students from metropolitan schools (89.98% *v*. 42.17%). Although one may presume that intervention schools were more well-resourced than control schools, and that the observed effect may instead be attributable to this, our findings indicate that the intervention schools in our sample were from less-well-resourced areas, including a higher proportion from lower socioeconomic, rural and remote settings. Nonetheless, we have attempted to counteract these two limitations by controlling for plausible confounders including grade, gender, socioeconomic and rurality status. A third limitation is the low Cronbach’s alpha observed for the CSI disengagement subscale in our sample, indicating that this finding should be interpreted with caution. Given that the reliability and validity of the scale have been demonstrated elsewhere,^26,27^ the low internal consistency observed in our study may be explained by the use of a sample with different sociocultural characteristics, different contextual factors or adaptation of the original scale. A fourth limitation is that owing to the anonymous nature of the survey and collection of data at one time point (post-test), the collected responses only represent those students who were present and willing to complete the survey on the day. We were thus unable to explore the characteristics of non-responders and the potential impact of response bias, implementing complete-case analysis to analyse the data. Our data-set also did not specifically identify students with special needs or those whose first language was not English. However, the survey providers have confirmed that teachers provided support to students affected by these circumstances to assist them in completing the survey. A final limitation is that data on ‘non-standard’ participants, who had attended their index school for a period of time shorter than that for which the programme had been implemented, were not collected. However, we note that this would be likely to bias the results in a more conservative direction.

The strengths of our study include its large sample size and inclusion of intervention schools that had implemented the programme for a duration anywhere from 2 to 8 years. This enabled identification of an important relationship between implementation duration and programme effectiveness, which warrants further investigation through robust longitudinal studies. We suggest that possible explanatory factors raised by our conceptual model could also be explored, for example: (a) through collection of data on how long individual students have received the programme and assessment of the subsequent impact on programme effectiveness; (b) by conducting a process evaluation to assess whether schools with longer implementation duration demonstrate higher-quality implementation and (c) by measuring school-level factors to assess whether schools with longer implementation duration demonstrate sustainable changes in school norms. Given the theoretical promise of whole-school initiatives, future research is required to further our understanding of the role of implementation duration in the success of these initiatives.

In summary, adolescents receiving The Resilience Project School Partnership Program demonstrated consistently better mental health outcomes than the control group, provided that schools had invested in the programme in the long term. We recommend that future research continue to investigate the importance of implementation duration in the success of whole-school initiatives, in addition to factors that may mediate this effect. Our study provides insights for stakeholders such as public health and policy makers, in particular, that whole-school approaches to mental health and well-being in adolescent populations may require long-term investment for their potential to be realised.

## Supporting information

Balasooriya Lekamge et al. supplementary material 1 Balasooriya Lekamge et al. supplementary material

Balasooriya Lekamge et al. supplementary material 2 Balasooriya Lekamge et al. supplementary material

Balasooriya Lekamge et al. supplementary material 3 Balasooriya Lekamge et al. supplementary material

Balasooriya Lekamge et al. supplementary material 4 Balasooriya Lekamge et al. supplementary material

## Data Availability

The data-sets analysed in the current study are not publicly available but are available from the corresponding author on reasonable request.
