# Supplementary material for: Evaluation of a nationwide whole-school approach to mental health and well-being in 40 149 Australian secondary school students: cluster quasi-experimental study
Source: BJPsych Open. 2025 Mar 11;11(2):e47. doi: 10.1192/bjo.2024.843 (PMC12001954; doi:10.1192/bjo.2024.843)
Supplement: Balasooriya Lekamge et al. supplementary material 1 — Balasooriya Lekamge et al. supplementary material [file S2056472424008433sup001.docx]

The intervention has been described in accordance with the TIDieR checklist.

| Item Number | Item |
| --- | --- |
| **Brief Name** | |
| 1 | The Resilience Project’s (TRP) School Partnership Program |
| **Why** | |
| 2 | Informed by Positive Psychology and Resilience Theory, the intervention adopts a whole-school approach to build resilience and happiness in young people through increasing the understanding and practice of gratitude, empathy, emotional literacy and mindfulness (GEEM). |
| **What** | |
| 3 | Schools were provided hard copies of and access to an online hub which included a guide for school ambassadors, a guide for teachers, a guide on how to engage parent and carer communities, resources for professional development and resources for curriculum mapping. These resources were intended to upskill education staff for the delivery of the intervention and to facilitate the practice of GEEM as a staff cohort. Digital student presentations, which were to be delivered as part of the curriculum, alongside 50-year-level-specific lessons plans were included on the online hub provided to schools. Additional resources to reinforce the practice of GEEM through the physical environment and activities outside of the curriculum were included in this online hub. Students received journals with individual activities to complement lessons in the curriculum. Parents and carers were provided access to an online hub which included articles and videos to increase their understanding of GEEM as well as family wellbeing activities to practice GEEM as a family. |
| 4 | The materials described above were made available to each respective party. Education staff were encouraged to engage with the resources on the online hub to build capacity for intervention delivery and engage in professional development activities aimed to facilitate staff practice of GEEM. Teachers delivered the curriculum in-school hours, through a combination of showing digital presentations from the intervention providers, undertaking class-wide and small group practical activities, and guiding students to engage in individual activities through student journals. Teachers were encouraged to engage with additional resources to reinforce GEEM outside of the curriculum; for example, through GEEM-themed chats and GEEM awards to embed the practice of GEEM in the school ethos and environment. Schools introduced parents and carers to the intervention and provided them with access to an online hub. |
| **Who provided** | |
| 5 | A Schools Partnership Manager was allocated to each school participating in the intervention to assist the school’s leadership team in mapping the project in line with school strategic plan, to build capacity in those delivery the program, to support the delivery of the program and address any challenges that arose during implementation. The curriculum was delivered by teachers, however, the digital presentations included in the curriculum were pre-recorded presentations by The Resilience Project staff. Education staff were responsible for encouraging the practice of GEEM outside of the formal curriculum, through embedding GEM in the school ethos and environment. The online hub for parents and carers comprised of resources created by The Resilience Project staff. |
| **How** | |
| 6 | Delivery of the intervention for students included a combination of face-to-face and digital modalities; for example, digital presentations to be watched in class alongside practical activities. The practical activities consisted of both group-level activities and individual-level activities (for example, completion of corresponding activities in student journals). Online resources for parents and teachers were delivered digitally. |
| **Where** | |
| 7 | Intervention components at the curriculum, school ethos and environment levels were administered within a school setting and within school hours. Teachers and parents accessed resources in their own time. |
| **When and How Much** | |
| 8 | Curriculum-based components were recommended to be delivered as weekly 60-minute lessons or split into 3 by 20-minute lessons. School ethos and environment-based components were recommended to be delivered daily, but ultimately at the teacher’s professional discretion. |
| **Tailoring** | |
| 9 | The intervention can be tailored based on the preferences of the school, with the Schools Partnership Manager liaising with schools in this process. Details on how each school tailored the intervention was not collected. |
| **Modifications** | |
| 10 | Not applicable given the nature of the study. |
| **How well** | |
| 11 | Not assessed. |
| 12 | Not assessed. |
