# Supplementary material for: Evaluation of a nationwide whole-school approach to mental health and well-being in 40 149 Australian secondary school students: cluster quasi-experimental study
Source: BJPsych Open. 2025 Mar 11;11(2):e47. doi: 10.1192/bjo.2024.843 (PMC12001954; doi:10.1192/bjo.2024.843)
Supplement: Balasooriya Lekamge et al. supplementary material 2 — Balasooriya Lekamge et al. supplementary material [file S2056472424008433sup002.pdf]

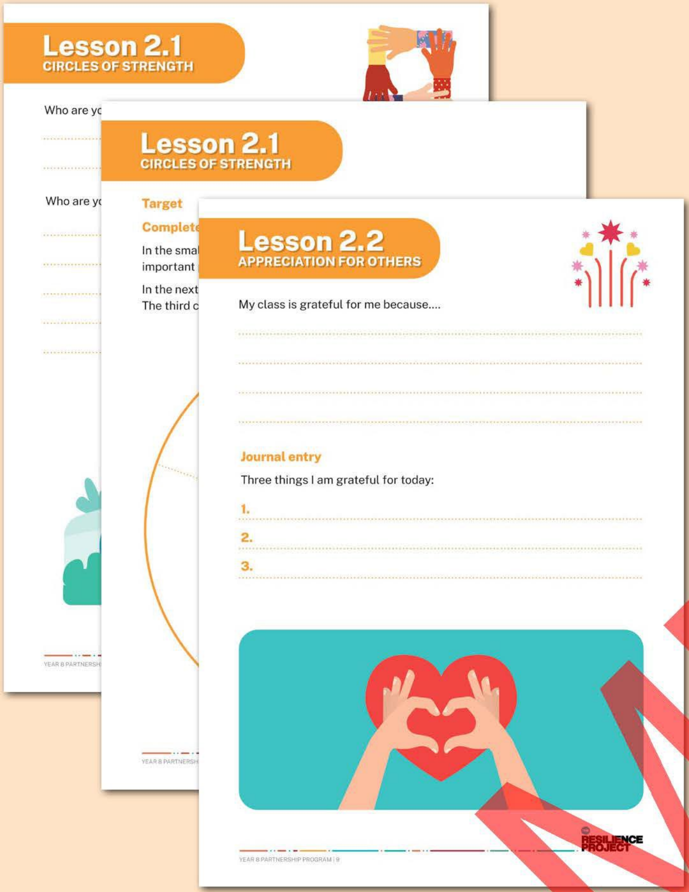

WEEKLY CHECKLIST

☐ GEM Chat

- Stick an A3 (or larger) sheet on the wall using Blu Tack or something similar.
- Ask each student to write something they are grateful for on the sheet, as they walk into the classroom.

☐ Habit Tracker Reminder

Don't forget to remind your students to fill in their weekly Habit Tracker and Mood Tracker in their student journals.

SAMPLE

Differentiation - Enabling Prompt: For **Activity 1** (Lesson 2.2), have a class discussion beforehand about the importance of kindness, and how a person would feel if they opened a piece of paper to read something that either was not overly positive or even empty. If needed, you can extend this discussion to how it may be 'funny' to them to do something like this now, but how would they and the person feel in the future? You can even highlight that in life (even as adults), there will be people we love to be around and others that we do not. However, it is important that we are kind and collegial. For example, not all teachers are friends outside of work, but they all work together as they want to achieve the best for themselves and their students. Just like this activity should be done properly for the benefit of the whole classroom.

Differentiation - Extending Prompt: For **Activity 1** (Lesson 2.1), have students share some of their responses with the group. You may get a lot of similar answers (i.e. friends, parents etc). Challenge them to think of less obvious people like their coach, teachers, celebrity etc.

If you have more time: Snowball fight continued (10 - 20 mins)

- Keep going with the snowball activity until everyone in the class has written one thing for each member of the group.
- Have students reflect in silence about the things that were written on their sheet. Get students to make a note of the common things that came up on their sheet in their Student Journals.

© The Resilience Project 2024

Lesson 2.1

STRENGTH : Circles of Strength

LEARNING INTENTIONS:

- Students will recognise the people around them who provide great support.

RESOURCES:

- Teacher Resource Hub.
- Student Journals.
- Internet access, speakers, projector.

OUTLINE:

Activity 1: Circles of strength (20 mins)

- Ask students to consider the strengths of the people around them and how they help them to be stronger.
- Ask your students to take a moment to answer the questions in their Student Journals:
  - Who are your support crew?
  - What do they give you?
- Students are to complete the 'target' in their Student Journals. In the smallest circle, students name five people they recognise as important people to them and a source of support to them. In the next level, students write the strengths that they recognise in each person. In the third circle, students write down the reason they are grateful for each person.

Lesson 2.2

APPRECIATION FOR OTHERS: Snowball Fight

LEARNING INTENTIONS:

- Students will be recognised and appreciated within the class.

RESOURCES:

- Teacher Resource Hub.
- Student Journals.
- Internet access, speakers, projector.

OUTLINE:

Activity 1: Snowball Fight (15 mins)

- Clear a space. Have each student write their name on a piece of paper, and the phrase, 'My class is grateful for me because...'
- Scrunch the pieces of paper up and have a snowball fight. When you yell 'freeze' the students must freeze. Now ask the students to pick up a piece of paper. They open up the paper and write one reason they are grateful for that person. For example:
  - Alex: My class is grateful for me because: you are always cheerful and go out of your way to check on how others are feeling.
- Repeat this process two more times.
- Ask students to find their paper following the conclusion of the activity. Students are then to record what their classmates have said about them in their Student Journals.

Please note: This activity can make some students quite vulnerable. When done properly, with supervision, the activity is very powerful, but it needs to be monitored properly. Ensure you, as the facilitator, are engaged with the task and make sure the statements being written are positive. Use your professional judgement to ascertain whether or not this will work with your group. If you believe it will not work, even after using the enabling prompt to help, complete the alternative task below.

Note: It is a great idea for you to participate in the snowball fight activity also.

Alternative task:

- Print out sheets of paper with the students' names on them. Play some reflective, quiet music and have students write gratitude notes for three people, ensuring distribution is equal.
- Share some with the class and discuss how it felt to be appreciated.

Activity 2: Reflective moments (5 mins)

- Using their Student Journals, ask students to answer the following question:
  - My three moments of awesome today are:

SAMPLE

© The Resilience Project 2024

Lesson 2.1
CIRCLES OF STRENGTH

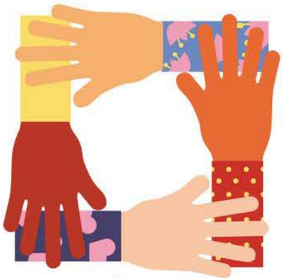

Who are you most grateful for?

Who are your support crew, and what do they give you?

SAMPLE

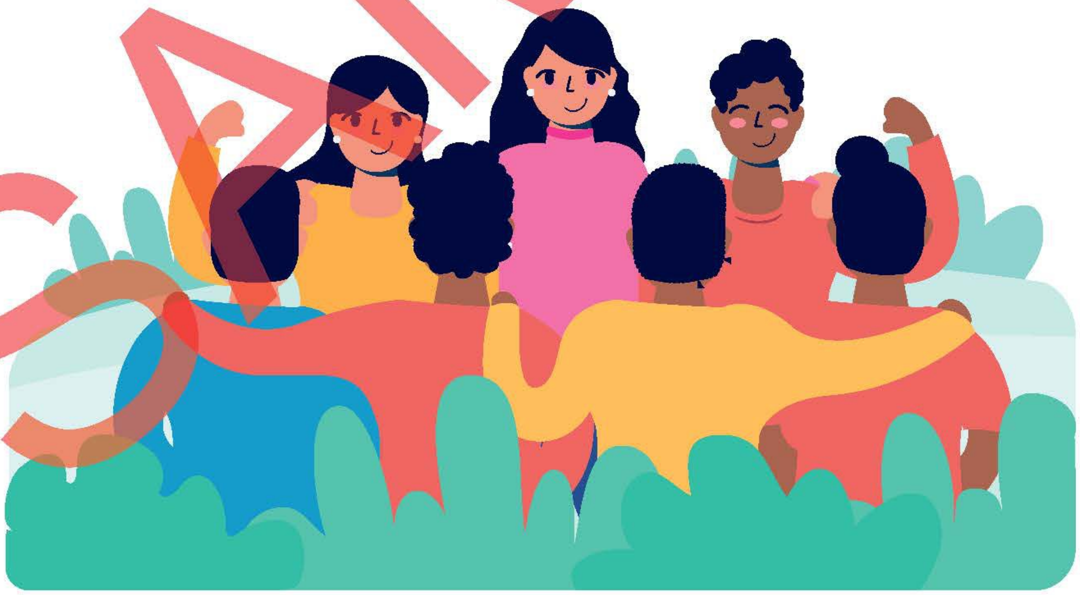

© The Resilience Project 2024

Lesson 2.1
CIRCLES OF STRENGTH

Target

Complete the 'target'.

In the smallest circle on the page, name **five people** you recognise as important people to you, and a source of support to you.

In the next level, write **the strengths** that you recognise in each person. The third circle is the reason **why you are grateful** for each person.

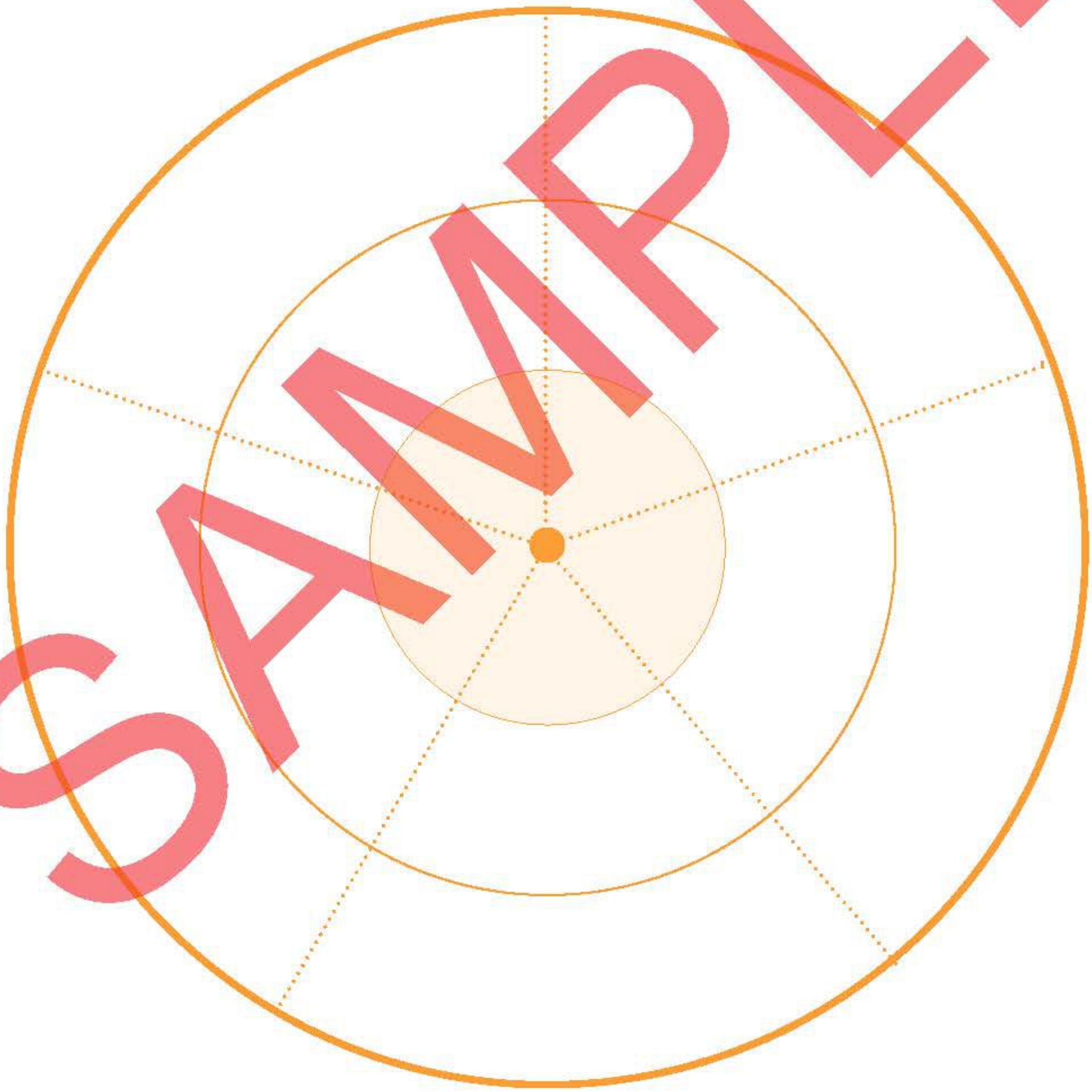

© The Resilience Project 2024

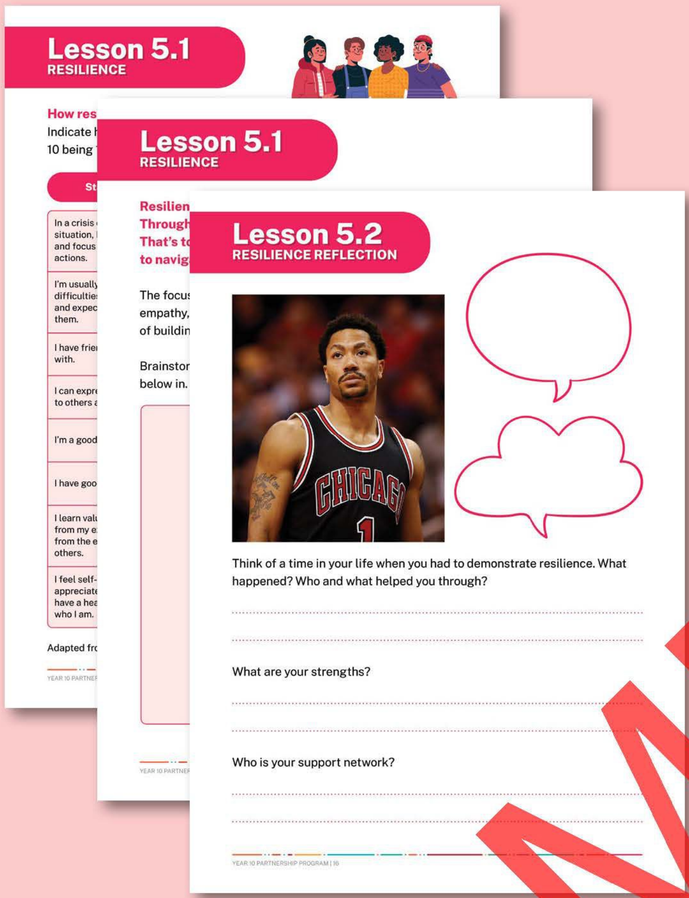

WEEKLY CHECKLIST

☐ GEM Chat

- Write the word 'resilience' in a cloud on an A3 sheet, the whiteboard or butcher's paper.
- Ask each student to write all the words they think of when they hear the world resilience, around the cloud.
- You can do this with a different word each week and put the finished posters all around the room. If you have coloured textas, you can use them to add colour to the poster.

☐ Habit Tracker Reminder

Don't forget to remind your students to fill in their weekly Habit Tracker and Mood Tracker in their student journals.

SAMPLE

Differentiation - Enabling Prompt: For **Activity 1** (**Lesson 5.1**), if students find the task too challenging to complete on their own, and if they are comfortable, ask them to pair up with another person. This will give them an opportunity to get some tips from others. Alternatively, you can give some general examples as a class. For example, if you wanted to improve in this area, what could you do?

Differentiation - Extending Prompt: For **Activity 1** (**Lesson 5.2**), have students share their responses with the class.

If you have more time: Share (10 mins)

- Divide students into groups of three and have them share the moment that required resilience that they recorded in their Student Journals.
- Compare support networks and strengths.

© The Resilience Project 2024

Lesson 5.1

RESILIENCE: How resilient are you?

LEARNING INTENTIONS:

- Students will understand the concept of resilience and apply it to their own situation.

RESOURCES:

- Teacher Resource Hub.
- Student Journals.
- Internet access, speakers, projector.

OUTLINE:

Activity 1: How resilient are you? Self evaluation (20 mins)

- Ask students to rate themselves for aspects of resilience using the quiz in their Student Journals. Explain that this is confidential and they will not need to share with the class.
- After completing the self-reflection, students need to pick three statements that were closer to 0 than 10 for them that they would like to improve.
- Ask students to brainstorm ways they could improve these areas in their Student Journals.

Lesson 5.2

RESILIENCE REFLECTION: The return

LEARNING INTENTIONS:

- Students will reflect on a time when they had to demonstrate resilience.

RESOURCES:

- Teacher Resource Hub.
- Student Journals.
- Internet access, speakers, projector.

OUTLINE:

Activity 1: The Return (15 mins)

- Watch **Video 1008**.
- Tell students to look for examples of what helped Derrek get through — positive self-talk, determination, family, friends, supporters.

Activity 2: Resilience reflection (5 mins)

- Ask students to open their Student Journals and complete the following questions:
  - > What are your strengths?
  - > Think of a time in your life when you had to demonstrate a great deal of resilience. What happened? Who and what helped you through?
  - > Who is your support crew?

SAMPLE

© The Resilience Project 2024

First Nations Cultural Connection

Discuss how your strengths can be used to serve and strengthen your community and the people around you. Identify what you believe is needed to improve the life of your community and how you could play a role in supporting others and places.

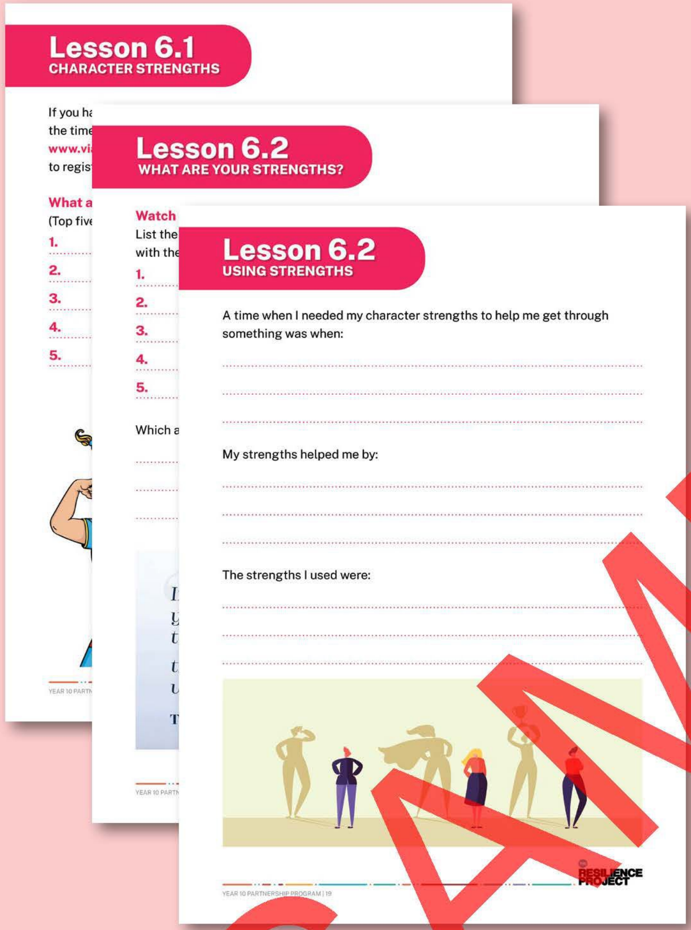

WEEKLY CHECKLIST

☐ GEM Chat

- For as many classes as possible this week, pick a character strength to focus on in that lesson. (You can use the character strength chart in the **Year 10 Teacher Resource Sheets** if needed).
- Write the strength on the board, get students to define it and give examples of classmates they have seen displaying that strength.

☐ Habit Tracker Reminder

Don't forget to remind your students to fill in their weekly Habit Tracker and Mood Tracker in their student journals.

SAMPLE

Differentiation - Enabling Prompt: For **Activity 2** (**Lesson 6.2**), use a character strength of your own to model to students how our character strengths can help us in different situations. For example, if someone has 'love of learning' as their character strength, then maybe they used the opportunity during Covid to teach themselves how to sew masks. Or if 'leadership' is their strength, perhaps they motivated their team during a game and their positive attitude helped win them the game.

Differentiation - Extending Prompt: For **Activity 1** (**Lesson 6.2**), have students identify the evidence of a growth mindset in Turia's story. How would her story look/sound like if she had a fixed mindset?

If you have more time: Character strength video (20 mins)

- Watch **Video 1032**.
- Have students identify two of their character strengths.
- Have students plan and record a video of each person in the class, highlighting their strengths. If you have the option, record this outside and in different settings.
- Alternatively, have students create an A3 poster of their top two (or chosen two) character strengths.

© The Resilience Project 2024

Lesson 6.1

CHARACTER STRENGTHS: Your strength

LEARNING INTENTIONS:

- Students will identify and explore character strengths in others.

RESOURCES:

- Teacher Resource Hub.
- Student Journals.
- Internet access, speakers, projector.
- Video/phone to record (If you have more time activity).
- Textas/pencils (If you have more time activity).
- A3 poster paper (If you have more time activity).

OUTLINE:

Activity 1: VIA Character Survey (25 mins)

- Explain to students that research has shown that using your character strengths can help you:
  - Buffer against, manage, and overcome problems.
  - Improve your relationships.
  - Enhance health and overall wellbeing.
- Have students take the time to complete the VIA Character Strengths Survey (for youth) at www.viacharacter.org
- It will take approximately 25 minutes. You will need to register on the website first.
- Character strengths are a great base from which to develop some common language and commonalities amongst the group.
- Ask students to open their Student Journals and record their top 5 from the VIA character Strength Survey. You will need to refer to these in other lessons.

Lesson 6.2

USING STRENGTHS

LEARNING INTENTIONS:

- Students will reflect on how their character strength has had a significant positive impact on their life.

RESOURCES:

- Teacher Resource Hub.
- Student Journals.
- Internet access, speakers, projector.

OUTLINE:

Activity 1: Turia — using her strengths (15 mins)

- Watch **Video 1010**.
- Ask students to list the character strengths Turia possesses, while they are watching the video.
- Share the lists of character strengths with the class.
- Have students reflect on which strength of Turia's they most admire and why.
- They can record their thinking in their Student Journals and then share with a partner, and then the class.

Activity 2: Using strengths (5 - 10 mins)

- Using the results of the VIA Character Strengths Survey — www.viacharacter.org, have students reflect on examples of when these strengths have helped them in their life. That strength may have helped them overcome a difficult time. Or it may be a positive experience that happened because of one of their character strengths.
- There is space in the Student Journals to record their thoughts.

SAMPLE

© The Resilience Project 2024

Lesson 5.1
RESILIENCE

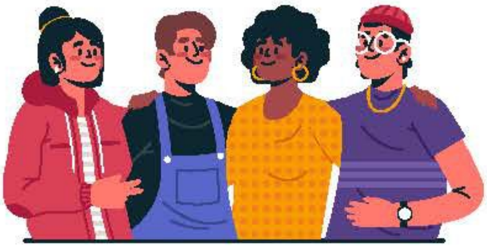

How resilient are you? Self evaluation

Indicate how much each statement applies to you. 1 being not at all (never), 10 being 100% me (all the time).

Statement	Rating									
In a crisis or chaotic situation, I calm myself and focus on taking useful actions.	1	2	3	4	5	6	7	8	9	10
I'm usually optimistic. I see difficulties as temporary and expect to overcome them.	1	2	3	4	5	6	7	8	9	10
I have friends I can talk with.	1	2	3	4	5	6	7	8	9	10
I can express my feelings to others and ask for help.	1	2	3	4	5	6	7	8	9	10
I'm a good listener.	1	2	3	4	5	6	7	8	9	10
I have good empathy skills.	1	2	3	4	5	6	7	8	9	10
I learn valuable lessons from my experiences and from the experiences of others.	1	2	3	4	5	6	7	8	9	10
I feel self-confident, appreciate myself, and have a healthy concept of who I am.	1	2	3	4	5	6	7	8	9	10

Adapted from the Resiliency Quiz - How Resilient Are You?, designed by Al Siebert, PhD.

SAMPLE

© The Resilience Project 2024

Lesson 5.1
RESILIENCE

Resilience is the ability to bounce back from challenges. Throughout the year, you are going to experience ups and downs. That's totally normal. It's important, however, that you know how to navigate your way through the tough times.

The focus of this course is to develop resilience by way of learning empathy, emotional literacy, mindfulness and gratitude — the cornerstones of building resilience.

Brainstorm ways you can improve in the areas your scored yourself 6 or below in.

SAMPLE

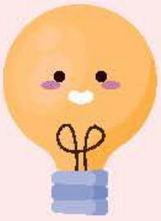

THE RESILIENCE PROJECT.

© The Resilience Project 2024

Lesson 5.2
RESILIENCE REFLECTION

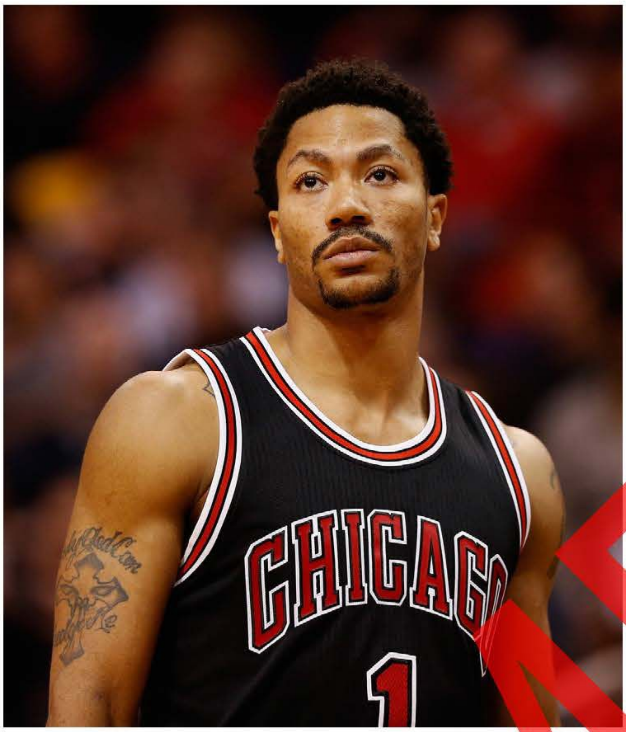

Think of a time in your life when you had to demonstrate resilience. What happened? Who and what helped you through?

....

....

What are your strengths?

....

....

Who is your support network?

....

....

SAMPLE

© The Resilience Project 2024

Lesson 6.1
CHARACTER STRENGTHS

If you have not already done so in the last year, it is recommended you take the time to complete the VIA Character Strengths Survey (for youth) at **www.viacharacter.org** It will take approximately 25 minutes. You will need to register on the website first.

What are your character strengths?

(Top five from the VIA Character Strengths Survey)

1. ....

2. ....

3. ....

4. ....

5. ....

Take it home: Tell your parents what your top character strengths are. Do they agree? Get them to complete the VIA Character Strengths if they haven't done so before.

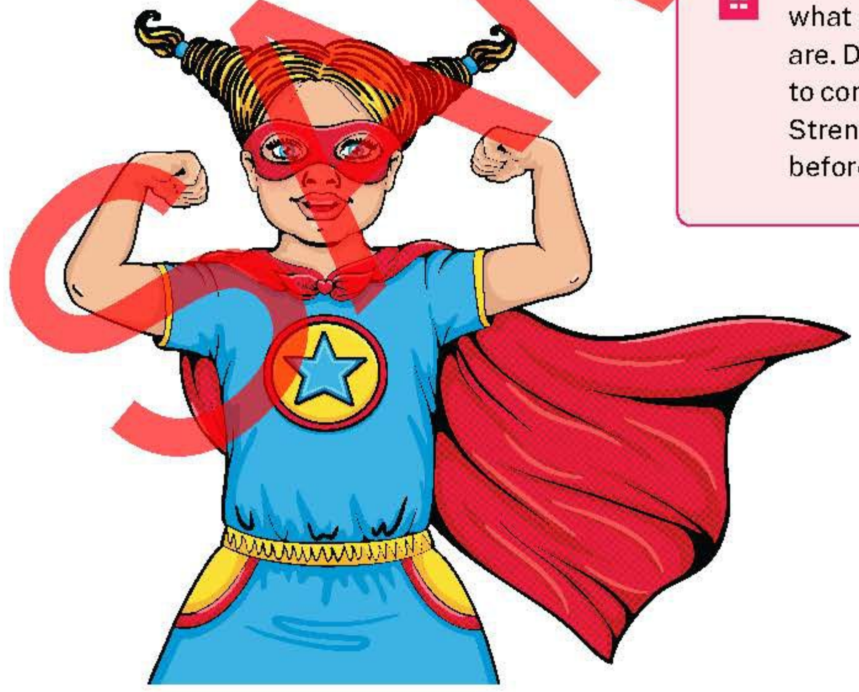

SAMPLE

© The Resilience Project 2024

Lesson 6.2
WHAT ARE YOUR STRENGTHS?

Watch the video about Turia.

List the character strengths that Turia possesses and share your responses with the class.

1. ....

2. ....

3. ....

4. ....

5. ....

Which are the character strengths in Turia that you most admire?

....

....

....

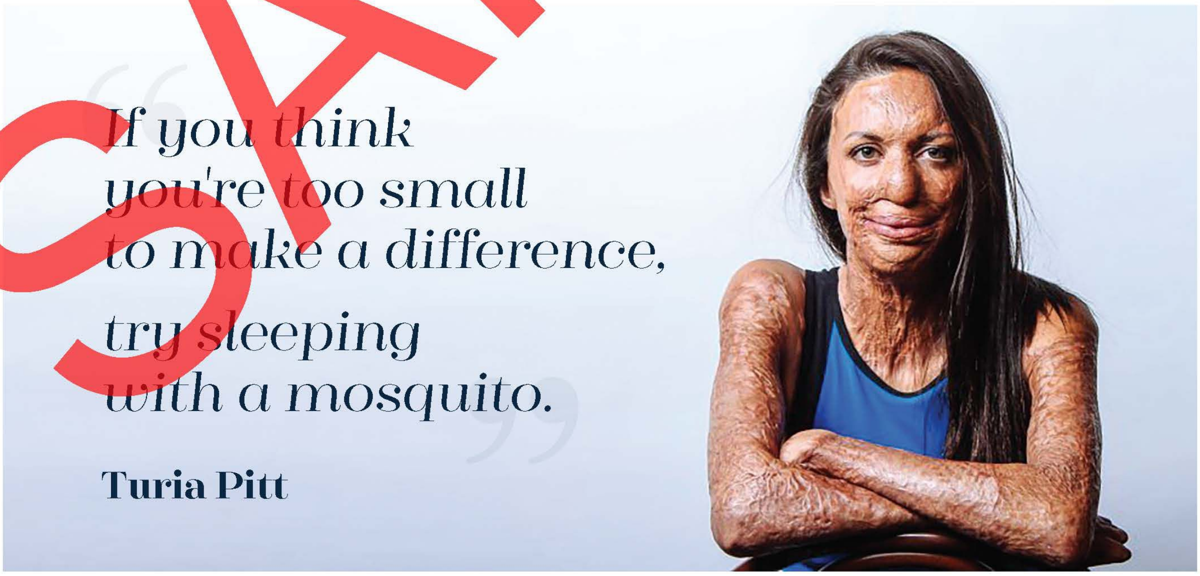

SAMPLE

© The Resilience Project 2024

Lesson 6.2
USING STRENGTHS

A time when I needed my character strengths to help me get through something was when:

My strengths helped me by:

The strengths I used were:

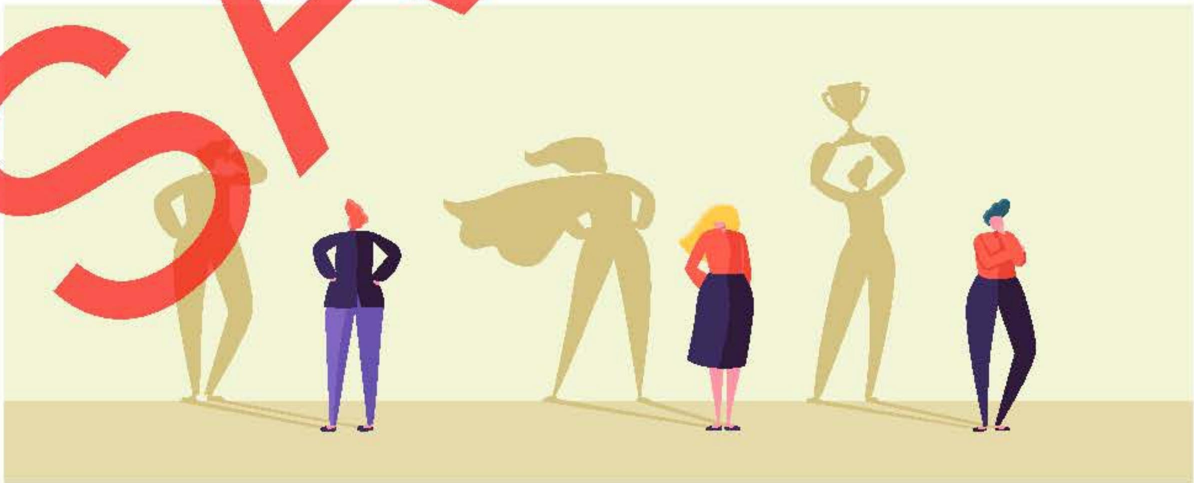

SAMPLE

© The Resilience Project 2024

First Nations Cultural Connection

Help students connect to their minds and bodies through deep listening. Listen to Dr Miriam-Rose Ungunmerr (AO), an Aboriginal Elder from Nauiyu (Daly River) speaking about "Dadirri" -Inner Deep Listening and Quiet Still Awareness, **Video 943**.

You can visit the Miriam-Rose Foundation to explore the teachings of Dadirri: **www.miriamrosefoundation.org.au/dadirri**

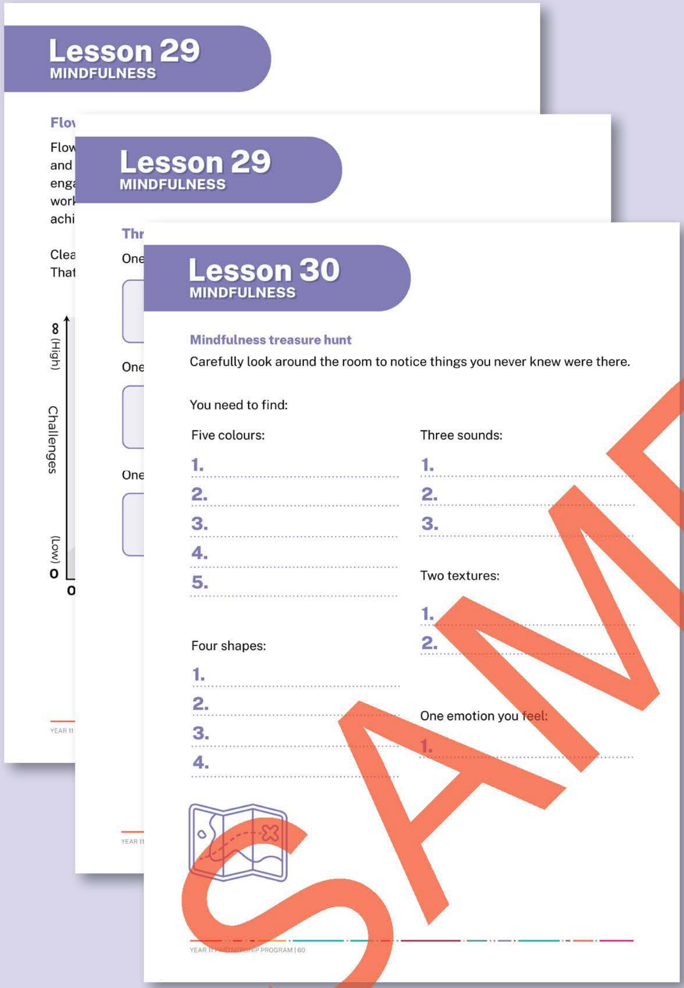

WEEKLY CHECKLIST

☐ **GEM Chat**

Remind your students to complete their GEM Habit Building Reflection pages in their Student Journals.

☐ **Habit Tracker Reminder**

Don't forget to remind your students to fill in their weekly Habit Tracker and Mood Tracker in their Student Journals.

If You Have More Time (5-10 mins)

Lesson 29

- Students could complete one of the reflections in their GEM Habit Building Reflection Pages in their Student Journals or complete their Weekly Habit and Mood Tracker.
- Encourage students to complete these reflections throughout the week, not just in class.

Lesson 30

- In pairs or small groups, have students share the following:
  - » Describe a moment when they felt completely present and connected today.
  - » Why and what did they notice?
  - » What would they like to do to be more present tomorrow?

© The Resilience Project 2024

Lesson 29 MINDFULNESS

LEARNING INTENTIONS:
- Students will explore mindfulness through flow.

RESOURCES:
- Student Journals.
- Balloons.
- Access to TRP Teacher Resource Hub.

OUTLINE:

Activity 1: Balloons (15 mins)
- Warm up with a game of keeping the balloon up in the air. The aim of the game is to keep the balloon up in the air as long as possible within the group by touching, hitting and kicking it gently. Divide the class into two teams and see who can keep their balloon in the air the longest. After a couple of minutes, divide the class into smaller teams again to increase the challenge.
- Debrief about the game:
  > Who found it too hard and wanted to give up?
  > Who found it too easy and was bored?
  > Who found it a good balance between the two — a good level of challenge? Explain that these people may have experienced a moment of flow. Go through the flow channel (this should be quite familiar to students). A visual of the flow channel can be found in their Student Journals.
- Flow channel: Flow is the state of optimal experience, a rewarding balance of our skills and the challenges before us. It's the enjoyable experience of being deeply engaged in the moment, and it is during moments of flow that our best work is produced, our greatest ideas expressed and our most admirable achievements accomplished.
- Clearly, flow is a good thing, but finding and maintaining flow is tricky. That's where the Flow Channel comes in.

Activity 2: Journal reflection (5 mins)
Have students complete the following in their Student Journals:
- Three things I noticed today:
  > One about my body.
  > One about my environment.
  > One about my thoughts.

Lesson 30 MINDFULNESS

LEARNING INTENTIONS:
- Students will experience mindfulness through paying attention to things they normally wouldn't.

RESOURCES:
- Student Journals.
- Access to TRP Teacher Resource Hub.

OUTLINE:

Activity 1: Whiteboard (10 mins)
- For the warm up meditation, instruct students:
  > Close your eyes. Take three deep breaths, in through your nose and out through your mouth.
  Imagine there is a huge whiteboard in front of you. Pick up a marker, feel the weight of it in your hand, the cool of the plastic.
  Take the cap off the pen — it has a familiar scent. Slowly take notice of the thoughts that are coming into your head and write them up onto the board — anything that comes to mind, write it on the board in your mind. (PAUSE).
  Keep writing until there is no space left on the board at all. (PAUSE).
  Now put the lid back on the pen, pick up the eraser and carefully wipe away all the writing, all your thoughts and feelings. (PAUSE).
  Take a deep breath, and slowly and quietly come back into the room.

Activity 2: Mindfulness treasure hunt (10 mins)
- Students are to carefully look around the room to notice things they never knew were there.
- They are to find:
  > Five colours.
  > Four shapes.
  > Three sounds.
  > Two textures.
  > One emotion they feel.
- Students write these into their Student Journals.

SAMPLE

2024 - TRP - Year 11 - Lesson Plans - PACE.indd 59 28/07/2023 3:59:08 PM
© The Resilience Project 2024

First Nations Cultural Connection

Aboriginal and Torres Strait Islander peoples are the longest continued culture on Earth! This means that they lived through some tough times and changes, and most importantly, knew how to live harmoniously. Discuss with students how we can all learn from the practices of Aboriginal and Torres Strait Islander peoples. You may like to watch Kanyini together; it can be accessed here: **www.kanyini.com**

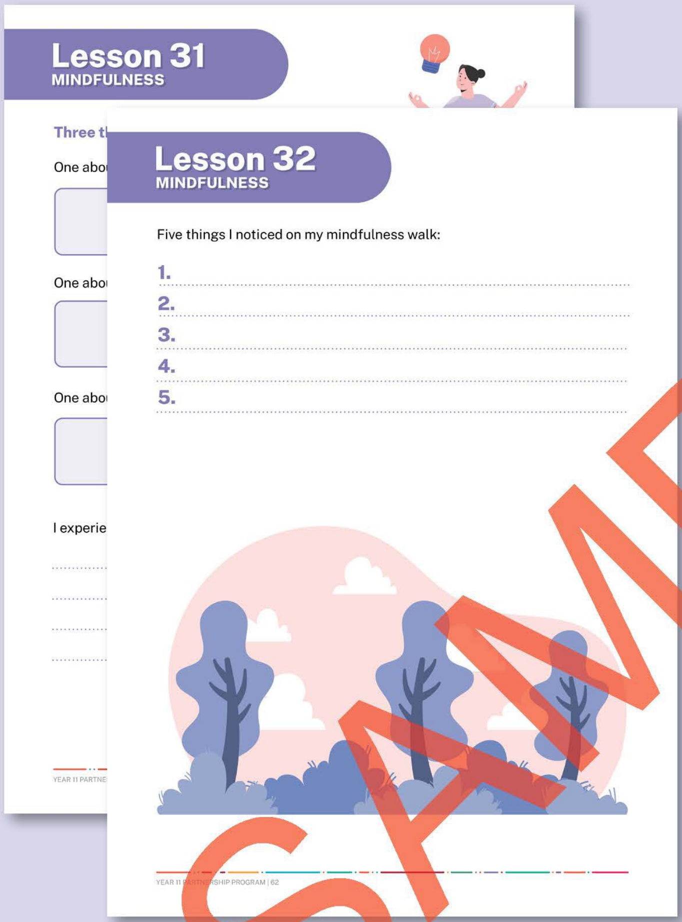

SAMPLE

WEEKLY CHECKLIST

☐ GEM Chat

Remind your students to complete their GEM Habit Building Reflection pages in their Student Journals.

☐ Habit Tracker Reminder

Don't forget to remind your students to fill in their weekly Habit Tracker and Mood Tracker in their Student Journals.

If You Have More Time (5-10 mins)

Lesson 31

- Ask students to think about their current habits. Do they have a morning routine? What about a pre bedtime routine?
- Ask students to identify some times in their current routine where they could add in some informal mindfulness (i.e. when brushing their teeth, having a shower, washing their hands).
- These moments can be used as a moment to be mindful, bringing all their awareness to that experience by noticing all 5 senses.
- This will help to create a habit of including mindfulness in their daily routine.

Lesson 32

- Students could complete one of the reflections in their GEM Habit Building Reflection Pages in their Student Journals or complete their Weekly Habit and Mood Tracker.
- Encourage students to complete these reflections throughout the week, not just in class.

© The Resilience Project 2024

Lesson 31
MINDFULNESS

LEARNING INTENTIONS:

- Students will experience mindfulness through mindful colouring.

RESOURCES:

- Student Journals.
- Colouring pencils, markers, textas.
- Access to TRP Teacher Resource Hub.

OUTLINE:

Activity 1: Colouring (15 mins)

- Play some music in the background: Video 1122. For the next 15 minutes, students are to colour-in a mindful colouring sheet at the back of their Student Journals.
- The purpose is for students to find themselves in flow and to be completely absorbed in the moment.

Activity 2: Journal reflection (5 mins)

Have students complete the following in their Student Journals:

- Three things I noticed today:
  > One about my body.
  > One about my environment.
  > One about my thoughts.
- I experienced 'flow' and being completely present in the moment when:

Lesson 32
MINDFULNESS

LEARNING INTENTIONS:

- Students will experience mindfulness in nature.
- Students will experience mindfulness through meditation.

RESOURCES:

- Student Journals.
- Access to The Resilience Project app or the Insight Timer meditation app/website.
- Access to TRP Teacher Resource Hub.

OUTLINE:

Activity 1: Mindfulness walk (10 mins)

- Go for a walk outside in silence. Challenge students to notice five things they would not normally notice.

Activity 2: Meditation (10 mins)

- Complete a meditation from The Resilience Project app or the Insight Timer meditation app/website that students have not heard recently.

SAMPLE

© The Resilience Project 2024
2024 - TRP - Year 11 - Lesson Plans - FA.indd 61 28/07/2023 3:59:14 PM

Flow channel

Flow is the state of optimal experience; a rewarding balance of our skills and the challenges before us. It's the enjoyable feeling of being deeply engaged in the moment, and it's during moments of flow that our best work is produced, our greatest ideas expressed and our most admirable achievements accomplished.

Clearly, flow is a good thing. But finding and maintaining flow is tricky. That's where the 'Flow Channel' comes in.

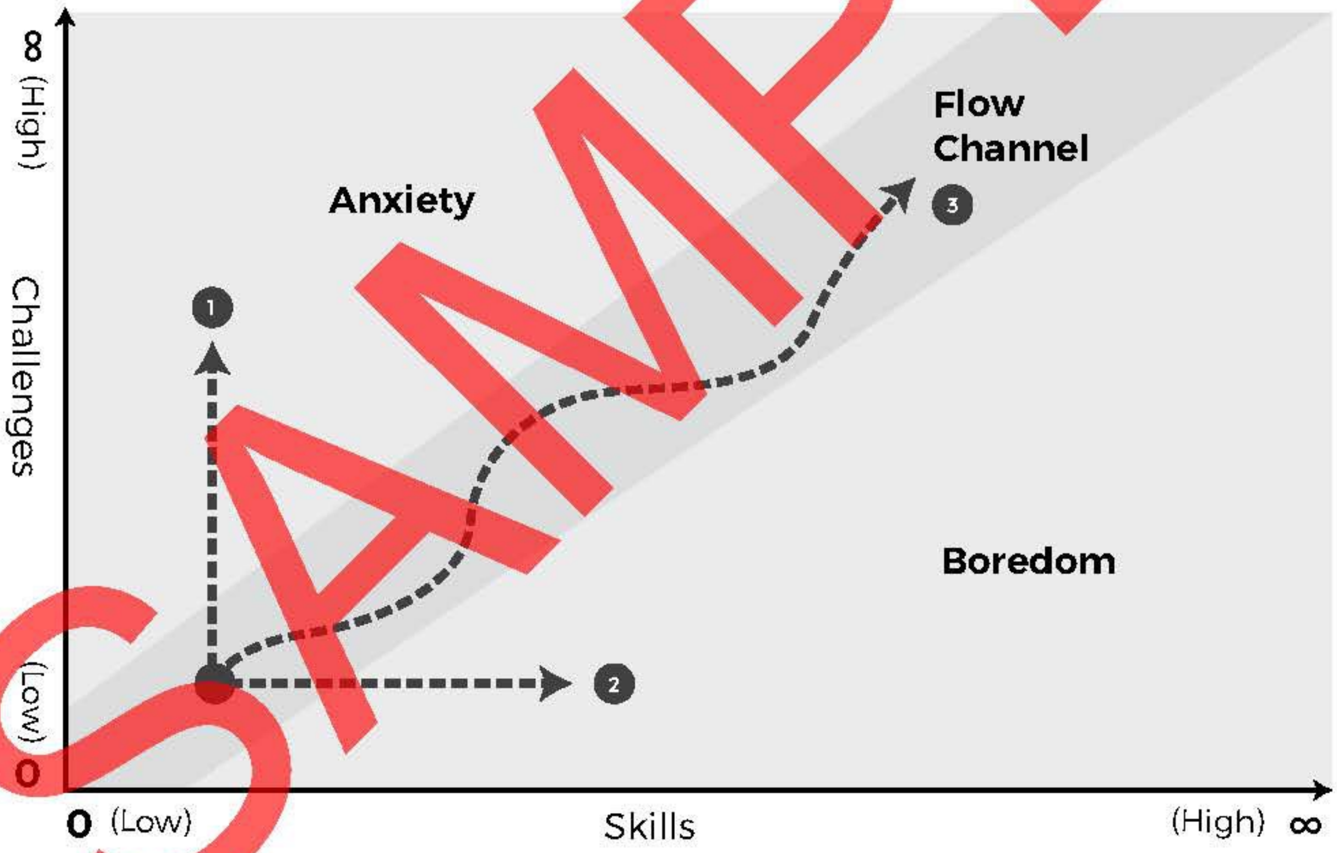

© The Resilience Project 2024

Lesson 29
MINDFULNESS

Three things I noticed today:

One about my **body**:

One about my **environment**:

One about my **thoughts**:

SAMPLE

© The Resilience Project 2024

Lesson 30
MINDFULNESS

Mindfulness treasure hunt

Carefully look around the room to notice things you never knew were there.

You need to find:

Five colours:

1. ....
2. ....
3. ....
4. ....
5. ....

Four shapes:

1. ....
2. ....
3. ....
4. ....

Three sounds:

1. ....
2. ....
3. ....

Two textures:

1. ....
2. ....

One emotion you feel:

1. ....

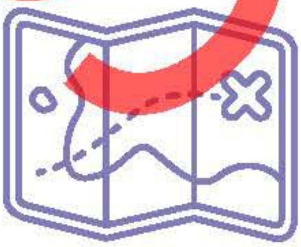

SAMPLE

© The Resilience Project 2024

Lesson 31
MINDFULNESS

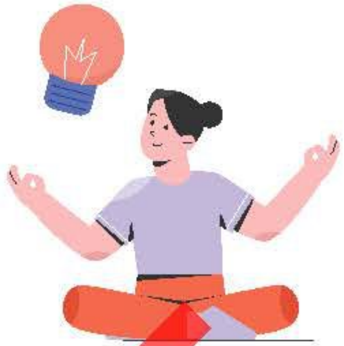

Three things I noticed today:

One about my **body**:

One about my **environment**:

One about my **thoughts**:

I experienced 'flow' and being completely present in the moment when:

SAMPLE

© The Resilience Project 2024

Lesson 32
MINDFULNESS

Five things I noticed on my mindfulness walk:

1.

2.

3.

4.

5.

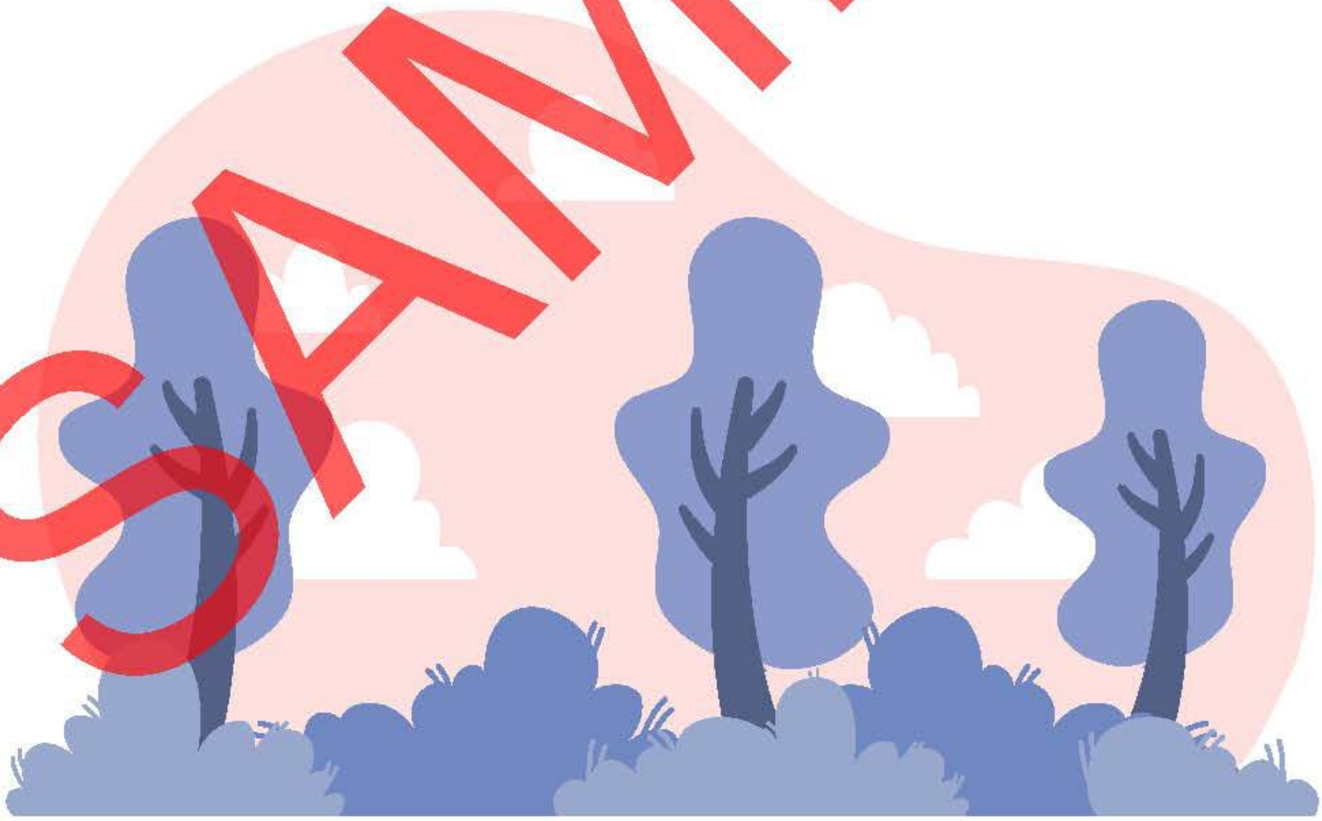

SAMPLE

© The Resilience Project 2024
