# Supplementary material for: Evaluation of a nationwide whole-school approach to mental health and well-being in 40 149 Australian secondary school students: cluster quasi-experimental study
Source: BJPsych Open. 2025 Mar 11;11(2):e47. doi: 10.1192/bjo.2024.843 (PMC12001954; doi:10.1192/bjo.2024.843)
Supplement: Balasooriya Lekamge et al. supplementary material 3 — Balasooriya Lekamge et al. supplementary material [file S2056472424008433sup003.docx]

Survey Questions for Mental Health Outcomes

Details about the complete Resilience Survey can be found here: <https://resilientyouth.org/survey>.

**Cantril Self-Anchoring Scale (CSAS)**

Question:

Imagine a ladder with steps numbered 1 at the bottom to 8 at the top. The top represents the best possible life for you and the bottom represents the worst possible life for you. On which step of the ladder do you feel you stand at this time?

Options:

1-8

**Children’s Hope Scale (CHS)**

Questions:

In most situations:

- Item 1: I think I am doing pretty well.
- Item 2: I can think of many ways to get things in life that are most important to me.
- Item 3: I am doing just as well as other kids my age.
- Item 4: When I have a problem, I can come up with lots of ways to solve it.
- Item 5: I think the things I have done in the past will help me in the future.
- Item 6: Even when others want to quit, I know that I can find ways to solve the problem.

Options:

6-point Liker scale: none of the time (1), a little of the time (2), some of the time (3), a lot of the time (4), most of the time (5), all of the time (6).

**Coping Strategies Inventory Disengagement Subscale (CSI)**

Questions:

- Item 1: When I have a problem, I avoid doing anything about it.
- Item 2: When I have a problem, I just wish it would go away.
- Item 3: When bad things happen, I usually blame myself.
- Item 4: When bad things happen, I talk to others about my problem.

Options:

4-point Likert scale: always or almost always (1), often (2), sometimes (3) and never or rarely (4).

**Generalised Anxiety Disorder (GAD-2)**

Questions:

Over the last 2 weeks, how often have you been bothered by the following problems?

- Item 1: Feeling nervous, anxious, or on edge.
- Item 2: Not being able to stop or control worrying.

Options:

4-point Likert scale: not at all (0), several days (1), more than half the days (2), nearly every day (3).

**Patient Health Questionnaire (PHQ-2)**

Questions:

Over the last 2 weeks, how often have you been bothered by the following problems?

- Item 1: Feeling down, depressed or hopeless.
- Item 2: Little interest or pleasure in doing things.

Options:

4-point Likert scale: not at all (0), several days (1), more than half the days (2), nearly every day (3).
