# Supplementary material for: Evaluation of a nationwide whole-school approach to mental health and well-being in 40 149 Australian secondary school students: cluster quasi-experimental study
Source: BJPsych Open. 2025 Mar 11;11(2):e47. doi: 10.1192/bjo.2024.843 (PMC12001954; doi:10.1192/bjo.2024.843)
Supplement: Balasooriya Lekamge et al. supplementary material 4 — Balasooriya Lekamge et al. supplementary material [file S2056472424008433sup004.docx]

Contents

[Life Satisfaction 3](#_Toc179194102)

[Control Versus Intervention Schools in their 2^nd^ or 3^rd^ Year of Implementation 3](#_Toc179194103)

[Control Versus Intervention Schools in their 4^th^ or 5^th^ Year of Implementation 4](#_Toc179194104)

[Control Versus Intervention Schools in their 6^th^ Year or Longer of Implementation 5](#_Toc179194105)

[Hope 6](#_Toc179194106)

[Control Versus Intervention Schools in their 2^nd^ or 3^rd^ Year of Implementation 6](#_Toc179194107)

[Control Versus Intervention Schools in their 4^th^ or 5^th^ Year of Implementation 7](#_Toc179194108)

[Control Versus Intervention Schools in their 6^th^ Year or Longer of Implementation 8](#_Toc179194109)

[Coping Skills 9](#_Toc179194110)

[Control Versus Intervention Schools in their 2^nd^ or 3^rd^ Year of Implementation 9](#_Toc179194111)

[Control Versus Intervention Schools in their 4^th^ or 5^th^ Year of Implementation 10](#_Toc179194112)

[Control Versus Intervention Schools in their 6^th^ Year or Longer of Implementation 11](#_Toc179194113)

[Anxiety 12](#_Toc179194114)

[Control Versus Intervention Schools in their 2^nd^ or 3^rd^ Year of Implementation 12](#_Toc179194115)

[Control Versus Intervention Schools in their 4^th^ or 5^th^ Year of Implementation 13](#_Toc179194116)

[Control Versus Intervention Schools in their 6^th^ Year or Longer of Implementation 14](#_Toc179194117)

[Depression 15](#_Toc179194118)

[Control Versus Intervention Schools in their 2^nd^ or 3^rd^ Year of Implementation 15](#_Toc179194119)

[Control Versus Intervention Schools in their 4^th^ or 5^th^ Year of Implementation 16](#_Toc179194120)

[Control Versus Intervention Schools in their 6^th^ Year or Longer of Implementation 17](#_Toc179194121)

# Life Satisfaction

## Control Versus Intervention Schools in their 2^nd^ or 3^rd^ Year of Implementation

## Control Versus Intervention Schools in their 4^th^ or 5^th^ Year of Implementation

## Control Versus Intervention Schools in their 6^th^ Year or Longer of Implementation

# Hope

## Control Versus Intervention Schools in their 2^nd^ or 3^rd^ Year of Implementation

## Control Versus Intervention Schools in their 4^th^ or 5^th^ Year of Implementation

## Control Versus Intervention Schools in their 6^th^ Year or Longer of Implementation

# Coping Skills

## Control Versus Intervention Schools in their 2^nd^ or 3^rd^ Year of Implementation

## Control Versus Intervention Schools in their 4^th^ or 5^th^ Year of Implementation

## Control Versus Intervention Schools in their 6^th^ Year or Longer of Implementation

# Anxiety

## Control Versus Intervention Schools in their 2^nd^ or 3^rd^ Year of Implementation

## Control Versus Intervention Schools in their 4^th^ or 5^th^ Year of Implementation

## Control Versus Intervention Schools in their 6^th^ Year or Longer of Implementation

# Depression

## Control Versus Intervention Schools in their 2^nd^ or 3^rd^ Year of Implementation

## Control Versus Intervention Schools in their 4^th^ or 5^th^ Year of Implementation

## Control Versus Intervention Schools in their 6^th^ Year or Longer of Implementation
